# Reasons for fear of cancer recurrence after endoscopic treatment of T1 esophageal adenocarcinoma. A semi-structured interview study

**DOI:** 10.1093/dote/doae067

**Published:** 2024-08-22

**Authors:** Wilda D Rosmolen, Roos E Pouw, Jacques J Bergman, Mirjam A G Sprangers, Pythia T Nieuwkerk

**Affiliations:** Department of Gastroenterology and Hepatology, Amsterdam UMC, University of Amsterdam, Boelelaan 1117, 1118, 1081HV Amsterdam, The Netherlands; Department of Gastroenterology and Hepatology, Amsterdam UMC, University of Amsterdam, Boelelaan 1117, 1118, 1081HV Amsterdam, The Netherlands; Department of Gastroenterology and Hepatology, Amsterdam UMC, University of Amsterdam, Boelelaan 1117, 1118, 1081HV Amsterdam, The Netherlands; Department of Medical Psychology, Amsterdam UMC, University of Amsterdam, Boelelaan 1117, 1118, 1081HV Amsterdam, The Netherlands; Department of Medical Psychology, Amsterdam UMC, University of Amsterdam, Boelelaan 1117, 1118, 1081HV Amsterdam, The Netherlands

**Keywords:** early esophageal cancer, endoscopic resection, psychology

## Abstract

Prior research has shown that patients with early Barrett’s neoplasia treated endoscopically report at least the same level of fear for cancer recurrence as patients treated surgically for a more advanced disease stage. The aim of this qualitative study was to gain insight into the reasons why endoscopically treated patients fear or not fear cancer recurrence. Patients treated endoscopically for T1 esophageal adenocarcinoma participated in a semi-structured interview. Patients were asked open questions about their fear of cancer recurrence and presented an a priori list of possible reasons for experiencing or not experiencing fear of cancer recurrence. Data saturation was reached with 12 patients who added 7 new reasons. Reasons that induced fear of cancer recurrence were related to physical symptoms, if cancer was diagnosed as an accidental finding and experiences with cancer in close relations. Endoscopic surveillance was mentioned as a reason for not experiencing fear of cancer recurrence. Patients reduced their fear of cancer recurrence by talking to close relations and seeking distraction. Caregivers reduced patients fear of cancer recurrence by giving adequate information and by showing photo of the treatment and the results of the treatment. According to patients with early Barrett’s neoplasia, receiving comprehensible information about the risk of recurrence and potential symptoms that may or may not be indicative of cancer recurrence, and continuing endoscopic surveillance, reduced fear of cancer recurrence. We recommend that healthcare providers discuss fear of cancer recurrence with their patients to enable tailoring information provision to their needs.

## INTRODUCTION

Barrett’s esophagus is a pre-malignant condition of the distal esophagus, which can lead to esophageal adenocarcinoma.^1–3^ Surgical treatment for esophageal adenocarcinoma is invasive with major anatomical changes of the upper gastrointestinal (GI) tract, a serious risk for post-surgical complications and mortality,^4^ impaired quality of life, and presence of long-term side effects.^5^ Endoscopic treatment with endoscopic resection with/without additional ablation of any residual Barrett mucosa at risk for progression is an excellent alternative for surgical treatment for patients with stage T1a and low risk T1b esophageal adenocarcinoma. Endoscopic treatment is very effective, minimally invasive, and organ sparing with excellent outcomes also on the long term.^6–9^ In a recent Dutch registry study including 1154 patients treated endoscopically for Barrett’s esophagus with early neoplasia, the risk for recurrence was only 3% after a median of 43 months follow-up.

Given the low risk of lymph node metastasis,^10^^,^^11^ local resection with multiband mucosectomy or endoscopic submucosal dissection are accepted alternatives to surgery for low-risk T1 cancer limited to the mucosa or superficial submucosa (<500 μm) without lymphovascular invasion and with well to moderate differentiation. For high-risk T1 cancer, the risk of lymph node metastases increases and surgical treatment is advised in current guidelines. However, in patients who are inoperable or refuse surgery, a watchful waiting policy may be applied.^11–13^

With the rising number of cancer survivors, the number of cancer survivors who report fear of cancer recurrence is also increasing, with prevalences ranging from 39% to 97% for any level of fear of cancer recurrence.^14^ Important risk factors for fear of cancer recurrence are the presence of physical symptoms, female gender, and younger age.^15^ Prior research has shown that patients with high-grade dysplasia and early esophageal adenocarcinoma (low-risk T1 cancer) treated endoscopically report at least the same level of fear for cancer recurrence after treatment as patients treated surgically for a more advanced stage of esophageal adenocarcinoma.^16^^,^^17^ Whereas the need for extensive endoscopic follow-up is reported as a risk factor for fear of cancer recurrence in other patients,^18–20^ the reasons for experiencing fear of cancer recurrence in patients treated for early Barrett’s neoplasia are not well understood.

To the best of our knowledge, no study has been published that explored the reasons for having fear of cancer recurrence in patients treated endoscopically for early esophageal adenocarcinoma. A better understanding of why these patients experience fear of cancer recurrence is needed to improve patient information and care. The objectives of this qualitative study among patients treated endoscopically for T1 esophageal adenocarcinoma are threefold: to explore^1^ the reasons why they fear or do not fear cancer recurrence,^2^ what patients did themselves to reduce fear of cancer recurrence, and^3^ what caregivers (the medical team) did to reduce or worsen fear of cancer recurrence according to patients.

## METHODS

### Patients

We included patients who were treated endoscopically and were under endoscopic follow-up after endoscopic treatment for T1 esophageal adenocarcinoma in the Amsterdam University Medical Centers (AUMC), between January 2018 and December 2019. At the AUMC around 60–80 patients with early Barrett neoplasia are treated per year, of which some are followed at the AUMC; some are referred back to the referral center for follow-up. Patients needed to be able to speak and read Dutch and give written informed consent. Whereas esophageal adenocarcinoma is most prevalent in men between 60 and 80 years, we aimed to ensure heterogeneity among the group of interviewees, by selecting patients with varying age and sex (purposive sampling), based on information provided in the patients’ files. Furthermore, we selected patients with varying pathology results and follow-up duration.^1^ Upfront, 15 patients were selected; however, the sample size was based on the principle of data saturation; when in three consecutive interviews, no new reason for having or not having fear for cancer recurrence was given, data collection stopped.

### Ethical considerations

The Amsterdam UMC Committee of Medical Ethics exempted the study from further ethical review since the Medical Research Involving Human Subjects Act (WMO) does not apply.

### Procedure

Eligible patients were mailed information about the qualitative study and an informed consent letter. If patients indicated to be willing to participate, an appointment was made for a telephone interview. The interviews were conducted by a nurse practitioner who was part of the medical team and the main investigator (WR). The interviews were audio-recorded. The interview manual and audio recording were tested in a pilot study with three patients. No changes needed to be made, and these patients were therefore included in the main study.

### List of reasons

Patients may have diverse, but not an unlimited number of reasons for fearing or not fearing cancer recurrence. We therefore devised an a priori list of 15 reasons based on literature^19^^,^^21–23^ and clinical experience, categorized into reasons related to physical symptoms (3 reasons), endoscopic treatment (4 reasons), cancer as accidental finding without symptoms (5 reasons), and experiences with cancer in close relations (3 reasons). For each reason, patients were asked to indicate whether it applied to them with respect to either experiencing or not experiencing fear of cancer recurrence or both. To exemplify, ‘Are physical symptoms like heartburn, full stomach, stomach pain or belching a reason for having or not having fear of cancer recurrence?’ The full list of reasons is displayed in Table 3.

### Semi-structured interviews

The interviews started with the question whether the current timing was still convenient. Patients were again assured that the content of the interview was confidential. Furthermore, the interviewer emphasized that the aim of this study was to explore patient’s experiences and that there were no right or wrong answers. The first three questions informed about marital status, education level, and employment status. Patients were then asked whether they currently experienced fear of cancer recurrence or not. Patients were further asked if they could give possible reasons for experiencing or not experiencing fear of cancer recurrence. After summarizing this information, patients were asked if they could think of more reasons for experiencing or not experiencing fear of cancer recurrence. Subsequently, each of the 15 reasons for experiencing or not experiencing fear of cancer recurrence was read aloud to the interviewees. After each reason, patients could indicate whether the reason applied to them and, if so, has led to experiencing fear of cancer recurrence, has not led to fear, or possibly both.

The subsequent question asked patients what they did to reduce possible fear (‘Did you do things yourself to reduce fear of cancer recurrence?’). The final four questions asked patients about the role of the caregivers in inducing or reducing fear of cancer recurrence (‘Did the caregivers say or do things that induced fear of cancer recurrence?; “Did the caregivers say or do things that reduced fear of cancer recurrence?’; ‘What could the caregivers have done to reduce fear of cancer recurrence?’; ‘What could caregivers do in the future to reduce fear of cancer recurrence?’).

### Worry of Cancer Scale

To assess a patient’s current level of fear of cancer recurrence, the interviewer read out loud the four-item Worry of Cancer Scale (WOCS).^24^ The questions were tailored to esophageal cancer. An example question is: ‘To what extent do worries about your esophageal illness dominate your thoughts and activities?’ The response options range from ‘certainly not’ (0) to ‘certainly’.^10^ The maximum score is 40, with a higher score indicating more worry.^24^ These questions were administered at the end to avoid their possible influence on the reasons given. The interview concluded with an invitation to make any comments a patient may have.

### Analysis

Content analysis was performed after each interview to identify new reasons for experiencing or not experiencing fear of cancer recurrence, using the audio recording. New reasons were added to the list. This extended list was used for the subsequent interview. Hence, patients who were interviewed later were invited to react to a longer list of reasons than those interviewed earlier. The final three interviews were those that would not yield new reasons for experiencing or not experiencing fear of cancer recurrence.

## RESULTS

### Patients

Between July 2019 and February 2021, 14 eligible patients who were treated for early esophageal adenocarcinoma were approached for the interview. The mean age of the participants was 70 years (range 49–82), and 75% (*n* = 9) of the participants were male. The mode for educational level was secondary vocational education (58%, *n* = 7). All but one patient were married. Eleven patients had one or more comorbidities. Each patient was treated with endoscopic resection. Two patients had an early cancer recurrence, again successfully treated with endoscopic resection. At the time of the interview, 8 of the 12 patients had or was still on additional treatment with radiofrequency ablation to remove the remaining Barrett’s epithelium. The histology of the resection specimen varied from low-risk T1 esophageal adenocarcinoma (<T1sm1N0M0, well to moderate differentiation, radically removed, no lymphovascular invasion) to high-risk T1 esophageal adenocarcinoma (T1sm2/3, and/or poor differentiation, and/or presence of lymphovascular invasion). The duration of follow-up ranged from 5 to 44 months (Table 1). After 12 interviews, data saturation was accomplished and data collection stopped.

**Table 1:** Patients’ characteristics at time of interview

Interview	Age (years)	Gender	Educational level	Marital status	Comorbidity	Time (months)	Remaining Barrett’s	Histology
1	72	Female	2	Widow	Yes	5	Yes	T1m3 G1 LV0 R0
2	49	Male	2	Married	Yes	22	No	T1m3 G1 LV0 R0
3	76	Male	2	Married	No	32	No	T1sm1 G1 LV0 R0
4	68	Male	2	Married	Yes	42	No	T1sm1 G1 LV0 Radically dubious
5	77	Male	3	Married	Yes	4	Yes	T1m3 G1 LV0 R0
6	82	Male	3	Married	Yes	10	Yes	T1sm2 G3LV0 R0
7	69	Male	2	Married	Yes	18	Yes	T1m3 G3 LV0 R0
8	76	Male	1	Married	Yes	44	No	T1sm2 G2 LV0 Radically dubious
9	66	Male	2	Married	Yes	42	No	T1m3 G3 LV0 R0
10	69	Female	3	Married	Yes	8	Yes	T1sm2 G3 LV0 R0
11	64	Male	2	Married	Yes	8	No	T1m3 G3 LV0 R0
12	76	Female	1	Married	Yes	5	Yes	T1sm2 G2 LV1 Radically dubious

Education level: 1: lower vocational education; 2: secondary vocational education; 3: higher vocational education.

Comorbidity: yes means at least one comorbidity. 1: hypertension; 2: obesity; 4: congenital hip dysplasia; 5: COPD, benign position vertigo; 6: hypertension, aneurysm abdominal aorta, atrial fibrillation, transient ischemic attack; 7: melanoma; 8: diabetes type II; 9: diabetes type II; 10: COPD, aorta dissection; 11: tuberculosis in childhood; 12: Chronic obstructive pulmonary disease (COPD), colon carcinoma, pulmonary embolism.

Time: between initial treatment and interview in months.

Remaining Barrett’s: residual Barrett’s epithelium at the time of the interview.

Radically dubious: histologically, dubious radically, endoscopically no sign of residual tumor.

### Interviews

The mean duration of the interviews was 27 minutes (range: 17–40 minutes). Seven reasons for experiencing or not experiencing fear of cancer recurrence were added to the original list of 15 reasons. The final list is shown in Table 3 with the patient numbers listed for each endorsed reason. The category ‘irrelevant’ was added to this table to indicate when a reason was not relevant for fear of cancer recurrence.

### Open question on worry about cancer recurrence

At the start of the interview, six patients reported not being worried about cancer recurrence. The other six patients indicated to be worried in different degrees.

### Reasons for experiencing or not experiencing fear of cancer recurrence

In response to the open question about possible reasons for having fear or not having fear of cancer recurrence, patients mentioned reasons that were included in the a priori list. The seven new reasons were suggested by three patients. Participating in the present qualitative study was the first new reason added to the list and was mentioned by one patient as a reason for experiencing fear of cancer recurrence. Additionally, information about the condition obtained from the internet or from the hospital were two additional reasons mentioned for having both fear and not having fear of cancer recurrence. Remaining Barrett’s epithelium and not being able to see what is happening inside of the esophagus were the fourth and the fifth new reasons mentioned for having fear of cancer recurrence only. To exemplify, patient 1 commented: ‘I can’t see how my esophagus looks from the inside and esophageal cancer gives symptoms in a late stage. Confidence in the medical team, the sixth reason, was both mentioned as a reason for experiencing fear and for not experiencing fear of cancer recurrence’. To exemplify: ‘I had full confidence in the medical team, because your team has a big name in the Netherlands for the endoscopic treatment of patients with early esophageal cancer. Even the specialized cancer hospital in the Netherlands send patients to your medical team for treatment’ (patient 9). The last reason added to the list was experiencing a spiritual event. This reason was mentioned by one patient and only as a reason for not experiencing fear of cancer recurrence. The patient explained: ‘Shortly after I was diagnosed with esophageal cancer and referred to the Amsterdam University Medical Centers, I was sitting in the garden with my wife, wondering if my cancer could be cured. At that moment, a butterfly came and sat on me for over 15 minutes. Then the butterfly flew away and came back three times to sit on my sweater for a brief moment. I told my wife: I don’t think we have to worry, everything will be alright’ (patient 9) and ‘because it took a long time before the pathology results were reviewed by an expert pathologist, I felt insecure and didn’t trust the outcome’ (patient 11).

### Final list of reasons

Patients most often indicated that 21 out of 22 reasons included in the list did neither induce nor reduce experiencing fear of cancer recurrence (i.e. irrelevant category). In total, 15 reasons of the final list were mentioned as a reason for fear of cancer recurrence and were related to physical symptoms, cancer as accidental finding without symptoms, and experiences with cancer in close relations. An interviewee explained as follows: ‘A friend of mine died from cancer, first they said the cancer was gone, but then it came back and he died. Therefore I am always a bit anxious that this could happen to me’ (patient 7). One reason, experiencing a spiritual event, was only mentioned as a reason for not experiencing fear of cancer recurrence. Four reasons were mentioned for both experiencing fear of cancer recurrence and not experiencing cancer recurrence. These reasons pertain to information obtained via internet or the hospital and confidence in the medical team. Endoscopic surveillance was primarily a reason for not experiencing fear of cancer recurrence. To illustrate: ‘I find the surveillance endoscopies reassuring. I can go on with my life’ (patient 5). ‘I feel safe due to the surveillance endoscopies’ (patient 7). ‘As time goes on and I have had more surveillance endoscopies, I become more reassured’ (patient 10). Only one patient indicated that surveillance induced fear: ‘Every three months I experience a lot of stress because I have to go for an endoscopy, then I have to wait for the results for two weeks. After that a period of relative peace follows and then it all starts over again’. Additional treatments and unclarity about what the treatment had done to the esophagus were not mentioned by any of the participants as reasons for either fear or not having fear for cancer recurrence (Table 3).

A notable finding is that remaining Barrett’s epithelium was mentioned as a reason for experiencing fear of cancer recurrence by three patients while these patients received radiofrequency ablation, where their remaining Barrett was removed. The four patients who did not report remaining Barrett’s epithelium as a reason for fear of cancer recurrence, still had remaining Barrett’s epithelium and had high-risk T1b esophageal adenocarcinoma (Table 2). Another finding worth mentioning is that three patients with low-risk T1 esophageal adenocarcinoma mentioned as reason for having fear of cancer recurrence that they were not adequately informed about their risk of cancer recurrence. None of the patients with high-risk T1 esophageal adenocarcinoma gave this as a reason for fear of cancer recurrence (Table 2).

**Table 2:** Overall overview of the results regarding fear of cancer recurrence

Patients		Experience FCR	Number of reasons for experiencing FCR	Number of reasons for not experiencing FCR	WOCS scores
1	F	Yes	10	2	10
2	M	No	0	1	0
3	M	No	3	1	5
4	M	No	1	1	6
5	M	Yes	8	1	18
6	M	No	0	0	8
7	M	Yes	5	1	7
8	M	No	0	1	0
9	M	No	3	1	0
10	F	Yes	2	1	5
11	M	Yes	7	0	27
12	F	No	0	1	14

FCR, fear of cancer recurrence; WOCS, Worry of Cancer Scale. Scores range from 0 to 40, with a higher score indicating more worry.

Experience FCR: open question at the start of the interview.

**Table 3:** List of reasons for fearing or not fearing cancer recurrence

Original questions	Reason for having fear of cancer recurrence	Irrelevant	Reason for not having cancer recurrence
1. Are physical symptoms like heartburn, full stomach, stomach pain, or belching a reason for having or not having fear of cancer recurrence?	1, 5, 9, 11	2, 3, 4, 6, 7, 8, 10, 12	
2. Are other physical symptoms a reason for having or not having fear of cancer recurrence?	5	1, 2, 3, 4, 6, 7, 8, 9, 10, 11, 12	
3. Is dysphagia a reason for having or not having fear of cancer recurrence?	5	1, 2, 3, 4, 6, 7, 8, 9, 10, 11, 12	
4. Are the additional treatments a reason for having or not having fear of cancer recurrence?		1, 2, 3, 4, 5, 6, 7, 8, 9, 10, 11, 12	
5. Is the fact that your esophagus is still present and the location where your cancer originated, reason for worrying or not worrying about cancer recurrence?	1, 5, 7	2, 3, 4, 6, 8, 9, 10, 11, 12	
6. Are regular surveillance endoscopies of the esophagus a reason for having or not having fear of cancer recurrence?	11	6	1, 2, 3, 4, 5, 7, 8, 9, 10, 12
7. Is unclarity about what the treatment does with your esophagus or has done for you a reason for having or not having fear of cancer recurrence?		1, 2, 3, 4, 5, 6, 7, 8, 9, 10, 11, 12	
8. Is uncertainty about the chance of cancer recurrence for you a reason to worry or not to worry about cancer recurrence?	1, 3, 5	2, 4, 6, 7, 8, 9, 10, 11, 12	
9. Is the accidental finding of the cancer without having symptoms a reason for having or not having fear of cancer recurrence?	7	1, 2, 3, 4, 5, 6, 8, 9, 10, 11, 12	
10. Is not sensing cancer recurrence a reason for having or not having fear of cancer recurrence?	1, 3, 7, 9	2, 4, 5, 6, 8, 10, 11, 12	
11. Is a family history of cancer a reason for having or not having fear of cancer recurrence?	1, 3, 11	2, 4, 5, 6, 7, 8, 9, 10, 12	
12. Is having another type of cancer a reason for having or not having fear of cancer recurrence?	1, 11	2, 3, 4, 5, 6, 7, 8, 9, 10, 12	
13. Are the worries of your family/friends about your esophageal cancer a reason for having or not having fear of cancer recurrence?	5, 7	1, 2, 3, 4, 6, 8, 9, 10, 11, 12	
14. Are the experiences with cancer among family or friends a reason for having or not having fear of cancer recurrence?	1, 5, 7, 10	2, 3, 4, 6, 8, 9, 11, 12	
15. Could worry about cancer recurrence help you to better deal with receiving such news?	4, 7, 10	1, 2, 3, 5, 6, 8, 9, 11, 12	
Added reasons			
16. Is this study a reason for having or not having fear of cancer recurrence?	1,	2, 3, 4, 5, 6, 7, 8, 9, 10, 11, 12	
17. Is information obtained from the internet about your condition a reason for having or not having fear of cancer recurrence?	11	2, 3, 4, 5, 6, 7, 8, 9, 10, 12	1
18. Is information obtained via the hospital about your condition a reason for having or not having fear of cancer recurrence?	11	2, 3, 4, 5, 6, 7, 8, 9, 12	1, 10
19. Is remaining Barrett’s epithelium a reason for having or not having fear of cancer recurrence?	1, 5, 9	2, 3, 4, 6, 7, 8, 10, 11, 12	
20. Is the fact that you cannot see what is happening to your esophagus a reason for having or not having fear of cancer recurrence?	1	2, 3, 4, 5, 6, 7, 8, 10, 11, 12	
21. Is confidence in your medical team a reason for having or not having fear of cancer recurrence?	11	8, 9, 12	7, 10
22. Is a spiritual event a reason for having or not having fear of cancer recurrence?		8, 10, 11, 12	9

An irrelevant category was added in this table to indicate when a reason was not relevant for fear of cancer recurrence.

### Worry of Cancer Scale

Three patients indicated not to worry (score 0). The other nine patients reported worry for cancer recurrence on at least one item of the WOCS to some extent, with their scores ranging from 5 to 27. Four patients who reported not fearing cancer recurrence at the start of the interview did report worry for cancer on the WOCS. The three patients who reported the highest scores on the WOCS also reported the highest number of reasons for having fear of cancer recurrence (>7 reasons) (Table 2).

### Additional questions

#### What patients did themselves to reduce fear

Seven patients mentioned the following practices: talking to family and friends, exercising, seeking distraction, carrying on with life, and consulting a psychologist. To illustrate: ‘I try to carry on with life by making plans, for example I planned a vacation and I signed up for a conference on an interesting topic’ (patient 5).

#### What did caregivers do that induced fear

Only one patient mentioned that the late availability of pathology results induced fear of cancer recurrence. The other 11 patients did not have any comment on this question.

#### What caregivers did to reduce fear

Nine patients indicated how their caregivers had reduced their fear of cancer recurrence, by giving adequate information about the condition and the treatment, being clear and honest, and, after the endoscopies showing photographs taken during endoscopic treatment, enabling the patients to monitor the progress of the treatment.

#### What caregivers could have done or could do to reduce fear

Four patients gave the following suggestions for the caregivers: use more comprehensible language, continue endoscopic surveillance, and organize a patient day with presentations and the possibility to meet fellow patients.

## DISCUSSION

To the best of our knowledge, this is the first qualitative study about fear of cancer recurrence among patients treated endoscopically for T1 esophageal adenocarcinoma. We aimed to investigate reasons why patients fear or do not fear cancer recurrence. Reasons that induce fear of cancer recurrence were physical symptoms, cancer as accidental finding without symptoms, and experiences with cancer in close relations. Endoscopic surveillance was mentioned as a reason for not fearing cancer recurrence. Furthermore, we found that patients reduced fear of cancer recurrence themselves by talking to family and friends, exercising, and seeking distraction. According to patients, caregivers reduced fear of cancer recurrence by giving adequate information about the condition and the treatment and by showing photographs of endoscopic treatment and the results of the treatment.

Barrett’s esophagus is associated with gastroesophageal reflux.^3^ Chronic reflux–related symptoms like heartburn, stomach pain, and belching are reasons for performing a diagnostic gastroscopy. A diagnosis of Barrett’s esophagus can be made during these endoscopies, and in some patients, early-stage esophageal adenocarcinoma is diagnosed while it is still asymptomatic, i.e. not causing the typical dysphagia complaints associated with more advanced esophageal adenocarcinoma. If patients experience reflux symptoms, they can unjustly relate these reflux symptoms to cancer. This may explain why some patients in the study mentioned reflux symptoms as a reason for fearing cancer recurrence. Patients have a tendency to focus on symptoms after cancer treatment, sometimes stimulated by caregivers who tell patients to be alert on alarm symptoms that may indicate cancer recurrence.^25^ Since patients cannot rely on symptoms (as they do not have them), it would have been advantageous if they could follow their own esophagus over time. However, since they cannot see inside their esophagus, this mere fact was also a source for experiencing fear of cancer recurrence as indicated by some patients.

Recurrence rates are very low in patients treated endoscopically for low-risk T1 esophageal cancer.^6^^,^^8^^,^^9^ Consequently, clinicians who treat these patients are increasingly convinced that extensive endoscopic surveillance after endoscopic treatment is not needed, because 100 follow-up endoscopies are required to find one recurrence, whereas the risk of mortality from other causes is 40 times higher than the risk of dying from esophageal cancer.^8^^,^^13^ Furthermore, clinicians assume that intensive endoscopic surveillance may induce fear of cancer recurrence, because patients are reminded of their esophagus every time they have to come to the hospital for an endoscopy and may fear the endoscopy itself as well as its results. However, the results of the present study do not support these assumptions; 10 out of 12 participants mentioned endoscopic surveillance as a reason for not experiencing fear for cancer recurrence. This reassuring effect of endoscopic surveillance may be explained by two reasons. First, literature shows that patients with fear of cancer recurrence may have the tendency to perform self-examination. However, self-examination to check esophageal cancer recurrence is not possible.^26^ Endoscopic surveillance and seeing pictures of treatment results are a possible alternative for self-examination and give patients a sense of security and control. Second, literature also shows that patients feel insecure, isolated, and alone when the contact with the medical team is reduced.^26–28^ Endoscopic surveillance ensures patients of regular contact with the medical team and hence provides reassurance.

Patient education about the chance of cancer recurrence after endoscopic treatment differs between patients with low- and high-risk T1 esophageal adenocarcinoma. Low-risk patients receive little, if any, information about their risk of loco-regional lymph node metastases. Endoscopic treatment is the gold standard for this specific cancer stage.^6^ Patients with high-risk stage 1 esophageal adenocarcinoma receive more extensive information about their risk of loco-regional lymph node metastases. Because of their higher risk, these patients can choose between standard surgical resection of the esophagus and strict endoscopic and endosonographic follow-up in a research setting. This may explain why three patients with low-risk T1 esophageal adenocarcinoma indicated not to know the chance of cancer recurrence as a reason for fear of cancer recurrence compared to none of the patients with high-risk T1 esophageal adenocarcinoma.

There are limitations of this study that merit attention. First, the number of patients included seems small. However, this number was sufficient to reach data saturation, a widely accepted methodological principle in qualitative research. Although we aimed for a heterogeneous group of patients, more men (nine) than women (three) participated in this study. The distribution between men and women, however, is conformed to clinical practice. Esophageal adenocarcinoma is most prevalent in men between 60 and 80 years.^1^ Third, the generalizability of the outcomes of this study might be limited due to the small patient sample and the fact that the study was conducted in a single hospital in the Netherlands, involving only patients of Caucasian ethnicity. However, it is worth noting that Barrett’s neoplasia is a disease predominantly found in individuals of Caucasian descent. Moreover, previous research has shown variable outcomes concerning ethnicity and the fear of cancer recurrence.^19^ Fourth, two of the reasons included in the a priori list were not mentioned by any of the patients as a reason for experiencing fear or not experiencing fear of cancer recurrence. This may suggest that these reasons are not applicable or that data saturation was not complete. Fifth, the interviewer is also part of the medical team. This may have induced socially desirable responses, particularly to questions regarding the caregivers. To minimize the effect of interviewer bias, patients were emphasized at the start of the interview that there were no right or wrong answers to the interview questions and that the purpose was to learn from patients’ experiences. Finally, the last five interviews were conducted during the first phase of the Corona pandemic, which may have influenced patients’ mental well-being and therefore their fear of cancer recurrence.

Patients treated for early esophageal adenocarcinoma indicate to worry and fear cancer (recurrence) as much as patients treated for advanced esophageal adenocarcinoma.^16^^,^^17^^,^^29^ Fear of cancer recurrence is a potential risk factor for impaired quality of life and increased healthcare costs.^30^ Fardell *et al*.^26^ suggest that caregivers should be alert to behavioral symptoms that can indicate heightened fear of cancer recurrence, such as self-focused attention, focus on symptoms, rumination, worries, and attempts to control or suppress thoughts about cancer recurrence.^26^ In daily practice, this means that caregivers should be alert to patients who frequently contact them, seek reassurance about their symptoms, frequently ask for endoscopic surveillance or other diagnostic tests like computed tomography scans, do not comprehend given information, and/or do not want to talk about what happened to them. When patients show one or more of the abovementioned behavioral symptoms, caregivers should address fear of cancer recurrence and emphasize that fear of cancer recurrence is widespread and normal.^30^ If necessary, patients should be advised to undergo psychological treatment, which is found to be effective in reducing fear of cancer recurrence.^26^^,^^31^^,^^32^ Furthermore, all patients treated endoscopically for early esophageal adenocarcinoma should be informed about the risk of cancer recurrence and about symptoms that may occur and which of these symptoms may or may not be indicative of cancer recurrence.^26^^,^^31^

Based on our findings, we recommend that healthcare providers discuss fear of cancer recurrence with their patients. This will enable them to tailor communication and information provision about risk of recurrence for patients with early Barrett’s neoplasia. This will prevent making assumptions about factors inducing or reducing fear of cancer recurrence in patients with early esophageal adenocarcinoma that are in discordance with patients’ opinions and experiences.

## Notes

Conflicts of interest: Roos. E. Pouw has received consultancy fee for MicroTech and Metronic and speaker fee from Boston Scientific and Pentax BV. Jacques J. Bergman has received unrestricted grants for research support from Cernostics, Olympus endoscopy, Aqua medical, and Medtronic; financial support for training programs from Medtronic and Pentax; consultancy fee from Olympus, Fractyl, Endogenex, Digma, and Aqua Medical; and study sponsoring from Fractyl laboraties, Endogenex, Digma, CDx Diagnostics, and Lucid.
